# The Value of Serum YKL-40 and TNF-*α* in the Diagnosis of Acute ST-Segment Elevation Myocardial Infarction

**DOI:** 10.1155/2022/4905954

**Published:** 2022-08-23

**Authors:** Caoyang Fang, Zhenfei Chen, Jing Zhang, Jianyuan Pan, Xiaoqin Jin, Mengsi Yang, Luyao Huang

**Affiliations:** ^1^Graduate School, Bengbu Medical College, Longzi Lake District, Bengbu, Anhui 233030, China; ^2^Department of Cardiology, The Second People's Hospital of Hefei, Hefei Hospital Affiliated to Anhui Medical University, Hefei, Anhui 230011, China

## Abstract

**Background:**

Acute ST-segment elevation myocardial infarction (STEMI) is a serious cardiovascular disease that poses a great threat to the life and health of patients. Therefore, early diagnosis is important for STEMI patient treatment and prognosis. The purpose of this study was to investigate the value of serum YKL-40 and TNF-*α* in the diagnosis of STEMI.

**Methods:**

From October 2020 to February 2022, 120 patients with STEMI were admitted to the Chest Pain Center of the Second People's Hospital of Hefei, and 81 patients with negative coronary angiography were selected as the control group. Serum YKL-40 and TNF-*α* concentrations were measured by sandwich ELISA. Pearson correlation was used to analyze the correlation between serum YKL-40, TNF-*α*, and serum troponin I (cTnI) in STEMI patients; multivariate logistic regression analysis was used to screen independent risk factors for STEMI. Three diagnostic models were constructed: cTnI univariate model (model *A*), combined serum YKL-40 and TNF-*α* model other than cTnI (model *B*), and combined cTnI and serum YKL-40 and TNF-*α* model (model *C*). We assessed the clinical usefulness of the diagnostic model by comparing AUC with decision curve analysis (DCA).

**Results:**

Serum YKL-40 and TNF-*α* in the STEMI group were significantly higher than those in the control group (*P* < 0.001). On Pearson correlation analysis, there was a significant positive correlation between serum YKL-40, TNF-*α*, and cTnI levels in STEMI patients. Multivariate logistic regression analysis showed that serum YKL-40 and TNF-*α* were independent risk factors for the development of STEMI. The results of ROC analysis showed that the area under the curve (AUC) of serum YKL-40 for predicting the occurrence of STEMI was 0.704. The AUC of serum TNF-*α* for predicting the occurrence of STEMI was 0.852. The AUC of cTnI as a traditional model, model *A*, for predicting the occurrence of STEMI was 0.875. Model *B* predicted STEMI with an AUC of 0.851. The addition of serum YKL-40 and serum TNF-*α* to the traditional diagnostic model composed of cTnI constituted a new diagnostic model; that is, the AUC of model *C* for predicting the occurrence of STEMI was 0.930. Model *C* had a better net benefit between a threshold probability of 70–95% for DCA.

**Conclusion:**

In this study, we demonstrate the utility of serum YKL-40 and TNF-*α* as diagnostic markers for STEMI and the clinical utility of diagnostic models by combining serum YKL-40 and TNF-*α* with cTnI.

## 1. Introduction

Acute ST-segment elevation myocardial infarction (STEMI) is a common cardiovascular disease in clinical practice, with high morbidity and mortality. Accurate diagnosis and treatment are helpful to reduce myocardial ischemic injury and reduce mortality [1]. Although electrocardiography is a common method for myocardial infarction, about 30% of patients do not have typical chest pain findings, and typical ST-segment changes characteristic of myocardial infarction can only be detected by electrocardiography at the onset. Therefore, relying only on clinical symptoms and electrocardiographic diagnosis easily leads to missed diagnosis and misdiagnosis. However, when the diagnosis of acute myocardial infarction is confirmed by coronary angiography, early treatment is often delayed and affects the prognosis [2, 3].

Serum YKL-40 is heparin- and chitin-binding glycoprotein secreted by macrophages, neutrophils, and vascular smooth muscle cells, which plays an important role in cell proliferation, differentiation, apoptosis, angiogenesis, tissue reorganization, and inflammation [4, 5]. Some studies [6] have found that YKL-40 is involved in the process of inflammation and vascular endothelial dysfunction and is closely related to atherosclerotic plaque formation. Similar to the above study, Batinic et al. [7] detected increased serum YKL-40 in patients with peripheral atherosclerosis, speculating that YKL-40 secreted by macrophages is involved in the formation and rupture of atherosclerotic plaques and that serum YKL-40 levels are closely related to the severity of atherosclerosis. Kucur et al. [8] found that serum YKL-40 levels were also increased in STEMI patients compared with healthy volunteers when serum YKL-40 concentrations were measured in patients with stable angina pectoris. Tumor necrosis factor-*α* (TNF-*α*) is an inflammatory factor with multiple biological activities secreted by activated monocytes and macrophages. It is an active and key component in the inflammatory response. The secretion of TNF-*α* in atherosclerotic plaques is mainly involved in the cascade of inflammatory responses, causes plaque rupture, and promotes thrombosis, promoting atherosclerotic progression. According to Kubenskii et al. [9] and Hou et al. [10], the serum TNF-*α* level was significantly higher in patients with stable exertional angina pectoris than in the control group and increased with the severity of coronary heart disease.

At present, there are few reports on whether serum YKL-40 and TNF-*α* have a correlation with STEMI occurrence and whether they can be biological markers to predict STEMI occurrence. This study aimed to investigate the diagnostic value of serum YKL-40 and TNF-*α* for STEMI and provide a reliable basis for the diagnosis and treatment of STEMI.

## 2. Materials and Methods

### 2.1. Study Population

*Clinical data.* From October 2021 to February 2022, 120 STEMI patients (93 males, mean age 61.65 years) admitted to the Chest Pain Center of the Second People's Hospital of Hefei were selected as the STEMI group. *Inclusion criteria*. STEMI diagnosis follows the diagnosis and treatment guidelines for STEMI [6] including [1] chest pain symptoms within 24 hours before admission, duration >30 minutes. [2] ECG showed ≥2 consecutive ST-segment elevations and/or abnormal *Q* wave new left bundle branch block; [3] serum biochemical markers such as creatine kinase isoenzyme (CK-MB) and/or cardiac troponin T (cTnT) increased within 24 hours after the onset of chest pain. Exclusion criteria are as follows: (1) history of PCI; (2) history of chronic heart failure, cardiomyopathy, valvular heart disease, arrhythmia, and congenital heart disease; (3) decompensated renal insufficiency; (4) history of chronic liver disease or liver dysfunction; (5) asthma, chronic obstructive pulmonary disease; (6) autoimmune disease and acute and chronic infection; and (7) patients with malignant tumors. Another 81 patients with negative coronary angiography during the same period were selected as the control group, including 63 males with a mean age of 62.25 years. The study was approved by the Ethics Committee of the Second People's Hospital of Hefei, and informed consent was signed by the patients or their families.

### 2.2. Study Methods

#### 2.2.1. Specimen Collection

We collect 5 ml of fasting cubital venous blood from STEMI patients before PCI and the control group in a common biochemical tube, centrifuge at 2500 r/min for 10 min at 4°C with a centrifugal radius of 15 cm, and separate the serum into a sterile EP tube, and store in a −80°C refrigerator until detection. Specimens were tested in the same batch and thawed fully and uniformly at room temperature for the first time.

#### 2.2.2. Basic Data

The baseline data of patients were recorded in detail through the hospital electronic case system, including age, gender, BMI, history of diabetes, history of hypertension, and history of smoking; all patients were sent for blood routine examination of venous blood within 24 hours after admission to detect white blood cells, hemoglobin, and platelets; biochemical indicators were measured on an empty stomach the next day, including serum total cholesterol, triglycerides, LDL-C, HDL-C, fasting blood glucose, serum creatinine, and blood uric acid.

### 2.3. Observation Indicators

Serum YKL-40 and TNF-*α* levels were measured by enzyme-linked immunosorbent assay (ELISA), and the kits were provided by Wuhan Huamei and Wuhan Ebotec Bioengineering Co., Ltd., China, and operated in strict accordance with the instructions.

### 2.4. Statistical Methods

SPSS 26.0 and *R* 4.2.1 statistical software were used for analysis. All measurement data were tested for normality. The normal distribution was expressed as mean ± standard deviation. An independent sample *t*-test was used for comparison between the two groups. The non-normally distributed measurement data were expressed as median *M* (*P*25, *P*75). Mann–Whitney *U* test was used for comparison. The adoption rate of enumeration data was expressed. The chi-squared test was used for comparison between the two groups. Pearson correlation was used to analyze the correlation between YKL-40, TNF-*α*, and high-sensitivity troponin I (cTnI). Multivariate logistic regression was used to analyze the risk factors of STEMI. The receiver operating characteristic curve (ROC) was used to evaluate the diagnostic value of serum YKL-40 and TNF-*α* for STEMI. Decision curve analysis (DCA) is to assess the clinical usefulness of diagnostic models. *P* < 0.05 was considered statistically significant.

## 3. Results

### 3.1. Comparison of Clinical Data between the Two Groups

**Figure 1:**
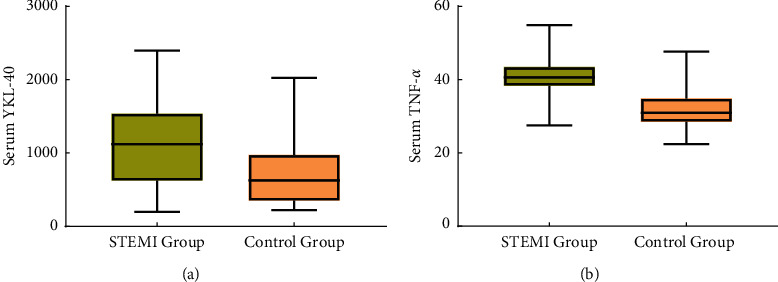
Data distribution of serum YKL-40 and TNF-*α* in two groups.

There was no significant difference in age, sex, history of hypertension, heart rate, hemoglobin, platelets, triglycerides, total cholesterol, LDL-C, creatinine, and uric acid between the two groups (*P* > 0.05). Two groups of patients were BMI, smoking history, diabetes history, systolic blood pressure, diastolic blood pressure, white blood cell, HDL-C, fasting blood glucose, LVEF, serum YKL-40, serum TNF-*α.* The differences in indexes were statistically significant (*P* < 0.05). As shown in Table 1, serum YKL-40 and TNF-*α*The data distribution in the two groups is shown in Figure 1.

### 3.2. Correlation Analysis of Serum YKL-40, TNF-*α*, and cTnI in STEMI Patients

**Figure 2:**
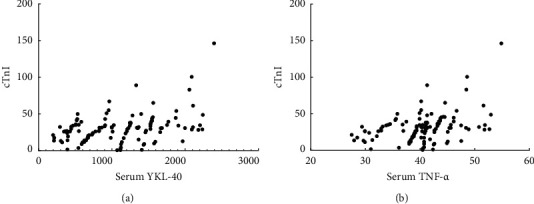
Correlation analysis of serum YKL-40, TNF-*α,* and cTnI.

Pearson correlation analysis showed that there was a significant positive correlation between serum YKL-40 and cTnI levels in STEMI patients (*r* = 0.405, *P* < 0.001) and serum TNF-*α*. There was a significant positive correlation with cTnI (*r* = 0.396, *P* < 0.001), as shown in Figure 2.

### 3.3. Analysis of Risk Factors for the Occurrence of STEMI

Univariate Logistic regression analysis showed that BMI, diabetes, smoking history, systolic blood pressure, diastolic blood pressure, white blood cell, HDL-C, fasting blood glucose, LVEF, serum YKL-40, and serum TNF-*α* were the risk factor for STEMI (*P* < 0.05). The traditional risk factors of STEMI, age, gender, and history of hypertension were also included in multivariate logistic regression analysis. The results showed that serum YKL-40 and TNF-*α* are an independent risk factors for STEMI (*P* < 0.05), as shown in Table 2.

### 3.4. The Value of Serum YKL-40 and TNF-*α* in the Diagnosis of STEMI

**Figure 3:**
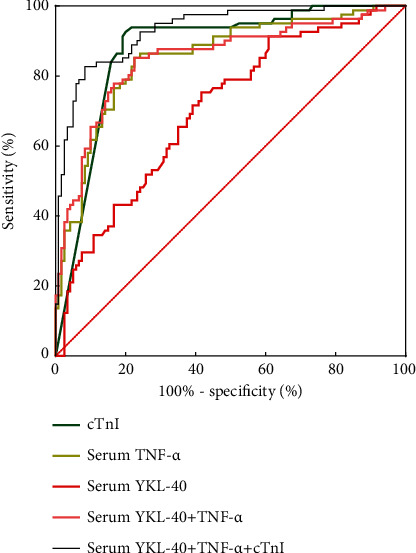
ROC curve of serum YKL-40 and TNF-*α* in the diagnosis of STEMI.

The ROC curve analysis showed that the area under the curve (AUC) of serum YKL-40 for predicting the occurrence of STEMI was 0.704 (95% CI: 0.632–0.776, *P* < 0.001), the sensitivity was 0.583, the specificity was 0.753, and the best cutoff value was 956.54 ng/dl. The AUC of serum TNF-*α* in predicting STEMI was 0.852 (95% CI: 0.797–0.907, *P* < 0.001), the sensitivity was 0.775, the specificity was 0.852, and the best cutoff value was 37.97 pg/ml. The AUC of cTnI as a traditional model, model *A*, for predicting the occurrence of STEMI was 0.875 (95% CI: 0.825–0.926, *P* < 0.001), with a sensitivity of 0.8 and a specificity of 0.926. Model *B* predicted STEMI with an AUC of 0.851 (95% CI: 0.794–0.908, *P* < 0.001), sensitivity of 0.775, and specificity of 0.852. The addition of serum YKL-40 and serum TNF-*α* to the traditional diagnostic model composed of cTnI constituted a new diagnostic model; that is, the AUC of model *C* for predicting the occurrence of STEMI was 0.930 (95% CI: 0.895–0.966, *P* < 0.001), with a sensitivity of 0.917 and a specificity of 0.827 as shown in Figure 3.

### 3.5. Decision Curve Analysis (DCA)

**Figure 4:**
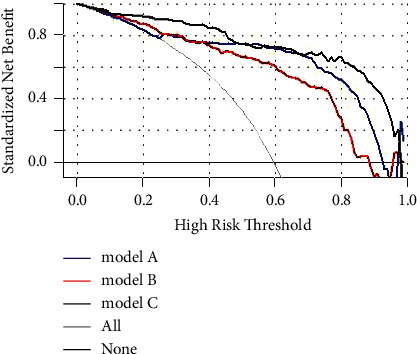
Decision curve analysis.

Figure 4 illustrates the decision curves for models A and B to predict the correct diagnosis of STEMI in patients. All models were effective between threshold probabilities of 60–90%, and model *C* had a better net benefit than model *A* between threshold probabilities of 70–95%.

## 4. Discussion

Acute ST-segment elevation myocardial infarction (STEMI) is a serious cardiovascular disease that poses a great threat to the life and health of patients [11]. At present, the early diagnosis of STEMI is mainly based on clinical symptoms of chest pain, ECG changes, and elevated myocardial injury markers [12]. Studies have shown that some STEMI patients have no typical early clinical symptoms, and myocardial injury markers are not significantly elevated. These characteristics lead to STEMI having a high rate of misdiagnosis and missed diagnosis, delaying the optimal timing of treatment for patients [13]. Therefore, how to diagnose STEMI early and time has become the focus of research, and it is particularly important to find biological markers with strong sensitivity and specificity for the diagnosis of STEMI.

The results of this study showed that there were significant differences in smoking history, diabetes history, and white blood cell ratio between the STEMI group and the control group; it indicated that smoking, blood glucose fluctuations, and inflammatory processes induced endothelial dysfunction, plaque formation to plaque instability, and destruction superimposed thrombosis, resulting in plaque rupture and inducing acute myocardial infarction leading to STEMI. In BMI, as a traditional risk factor for STEMI, this study showed that BMI was lower in STEMI patients than in controls, and the reason for analysis may be that the small sample size of this study has some bias on the study results. STEMI as a serious cardiovascular disease often leads to systemic hypoperfusion or even shock. The results of this study showed that systolic blood pressure, diastolic blood pressure, and LVEF in STEMI patients were significantly lower than those in the control group, indicating that myocardial cells were damaged or even necrotic heart pump function decreased after STEMI leading to decreased blood pressure or even shock; at this time, we need a specific and efficient diagnostic marker to early diagnose STEMI.

YKL-40, also known as chitinase-3-like protein 1 (CHI3L1), is a heparin- and chitin-binding glycoprotein mainly secreted by a variety of cells, including macrophages, chondrocytes, neutrophils cells, and vascular smooth muscle cells [4, 14]. As an inflammatory glycoprotein, YKL-40 is involved in endothelial dysfunction by promoting chemotaxis, cell adhesion and migration, reorganization, and extracellular matrix remodeling [5, 15]. YKL-40 induces the maturation of monocytes into macrophages in the early and late stages of the atherosclerotic process and is secreted by activated macrophages in the later stages of differentiation [16, 17]. Studies have shown that serum YKL-40 is involved in the formation of thin fibrous cap atheromatous plaques and plaque damage in diseased blood vessels in patients with coronary heart disease [18]. Studies have shown that elevated levels of YKL-40 have been detected in the serum of STEMI patients [19]. Similar to the above research results, the results of this study showed that the serum YKL-40 level in the STEMI group was significantly higher than that in the control group, and the difference was statistically significant (*P* < 0.05). It shows that serum YKL-40 is involved in the occurrence of STEMI, which may be related to the secretion of YKL-40 by macrophages in atherosclerosis and the release of YKL-40 when the atherosclerotic plaque ruptures, suggesting that serum YKL-40 may predict the occurrence of STENI important indicators.

Serum TNF-*α* is an inflammatory cytokine with more functions, which is generally secreted by activated macrophages and is closely related to the activity of immune response, inflammation, and related diseases. The formation and progression of thrombus are promoted [20]. In the generation and development of atherosclerotic plaques, TNF-*α* plays a great role in promoting and can break the balance of coagulation-fibrinolysis by inhibiting the synthesis of thrombomodulin (TM) by endothelial cells. This prevents TM from combining with thrombin in a timely and effective manner, thereby preventing the anticoagulant protein C from exerting its effect, and finally resulting in the generation of thrombus [21]. Normal cardiomyocytes cannot produce TNF-*α*, but in the event of pathological changes, cardiomyocytes produce a large amount of TNF-*α* mRNA and express TNF-*α* under the stimulation of various factors [22, 23]. Studies have shown that serum TNF-*α* in patients with acute myocardial infarction and unstable angina pectoris is significantly higher than that in normal control groups, especially in the acute myocardial infarction group, which indicates that TNF-*α* levels are related to the degree of myocardial ischemia [24]. In this study, ELISA was used to detect the serum TNF-*α* concentration of STEMI patients and control patients. The results showed that the serum TNF-*α* concentration of STEMI patients was significantly higher than that of the control group, suggesting that the level of TNF-*α* is closely related to the activation of atherosclerotic plaques. It indicates that TNF-*α* is involved in the occurrence and development of STEMI.

High-sensitivity troponin I (cTnI) is a reliable indicator for early diagnosis of acute myocardial infarction, disease monitoring, efficacy observation, and prognosis assessment [25]. To further explore whether serum YKL-40 and TNF-*α* levels are correlated with STEMI, Pearson correlation analysis showed that serum YKL-40 and TNF-*α* were positively correlated with high-sensitivity cTnI (*r* = 0.405, *P* < 0.001; *r* = 0.396, *P* < 0.001). After exclusion of traditional risk factors, age, gender, history of hypertension, and univariate analysis of statistically significant indicators were included in multivariate logistic regression analysis, serum YKL-40, and TNF-*α* were independent risk factors for STEMI (OR = 0.998, 95% CI: 0.995–1, *P*=0.038; OR = 1.496, 95% CI: 1.254–1.784, *P* < 0.001), further indicating that serum YKL-40 and TNF-*α* are related to the occurrence of STEMI and suggesting that serum YKL-40 and TNF-*α* can be used as a biological indicator for the diagnosis of STEMI.

To further explore the value of serum YKL-40 and TNF-*α* in the diagnosis of STEMI, the ROC curve analysis showed that the area under the curve (AUC) of serum YKL-40 for predicting the occurrence of STEMI was 0.704, the sensitivity was 0.583, and the specificity was 0.753. The area under the curve (AUC) of serum TNF-*α* in predicting STEMI was 0.852, the sensitivity was 0.775, and the specificity was 0.852. The area under the curve (AUC) of serum YKL-40 combined with TNF-*α* in predicting the occurrence of STEMI was 0.851 (95% CI: 0.794–0.908, *P* < 0.001), the sensitivity was 0.775, and the specificity was 0.852. The results of ROC analysis showed that the diagnostic value of serum TNF-*α* in STEMI was significantly higher than that of serum YKL-40 in sensitivity and specificity. And the combination of the two in the diagnosis of STEMI is the same as that of single serum TNF-*α* in the diagnosis, sensitivity, and specificity.

To compare with the traditional model composed of cTnI, the area under the curve (AUC) for predicting the occurrence of STEMI using the traditional model composed of cTnI as model *A* was 0.875 (95% CI: 0.825–0.926, *P* < 0.001), with a sensitivity of 0.8 and a specificity of 0.926. In the traditional diagnostic model composed of cTnI, serum YKL-40 and serum TNF-*α* were introduced to constitute the new diagnostic model, that is, model *C*. In the new diagnostic model, ROC results showed that the area under the curve (AUC) of the combination of the three to predict the occurrence of STEMI was 0.930 (95% CI: 0.895–0.966, *P* < 0.001), and the sensitivity was 0.917, and the specificity was 0.827. The AUC comparison between model *A* and model *B* was statistically significant at *P*=0.0059. The results showed that model *C* was superior to model *A* in the diagnosis of STEMI and had certain significance in guiding clinical work.

In parallel, we performed DCA to assess the performance of the diagnostic model. Model *C* had a net benefit better than model *A* with a threshold probability of 70–95%. It further illustrates that model *C* is superior to model *A* for STEMI diagnosis.

### 4.1. Limitations

First of all, this study is a single-center cross-sectional study with a small sample size, which will inevitably cause some biases to cause differences in results from previous studies. A multicenter, large-sample prospective study is required for further verification. Second, patients in the control group of this study were not tested for high-sensitivity cTnI, and the diagnostic value of serum YKL-40 and TNF-*α* and high-sensitivity cTnI in STEMI could not be compared in ROC analysis. Third, because this study was a single-center, small-sample study, no internal or external validation of the model was performed.

## 5. Conclusion

In conclusion, the results of this study showed that serum YKL-40 and TNF-*α* were significantly increased in the serum of STEMI patients, which were both related to the occurrence of STEMI and could be used as biological indicators for the occurrence of STEMI. Combined with the traditional diagnostic model cTnI, it had higher diagnostic value, stronger sensitivity, and specificity, and could provide a reliable basis for the diagnosis and treatment of STEMI.

## Figures and Tables

**Table 1:** Comparison of general clinical information between STEMI group and control group.

Variables	STEMI group	Control group	*t*/*χ*^*2*^/*z* value	*P* value
Age (years)	61.65 ± 13.66	62.25 ± 9.95	0.337	0.736
Gender (male, *n*%)	93 (77.5)	63 (77.8)	0.002	0.963
BMI (kg/m^2^)	24.44 ± 3.61	25.32 ± 2.92	2.052	0.041^*∗*^
Diabetes (*n*%)	37 (30.8)	10 (12.3)	9.226	0.002^*∗*^
Hypertension (*n*%)	68 (56.7)	46 (56.8)	0	0.986
Smoking (*n*%)	77 (64.2)	20 (24.7)	30.179	<0.001^*∗*^
Heart rate (beats/min)	80.88 ± 16.84	78.77 ± 15.86	0.895	0.372
Systolic pressure (mmHg)	124.91 ± 28	135.69 ± 18.69	3.038	0.003^*∗*^
Diastolic pressure (mmHg)	70 (60.25, 84)	81 (75.5, 90)	4.686	<0.001^*∗*^
Neutrophils (×10^9^/L), *M* (*P*_25_, *P*_75_)	10.44 (7.74, 12.64)	6.07 (4.88, 7.45)	9.056	<0.001^*∗*^
Hemoglobin (g/L)	137.02 ± 18.43	135.44 ± 13.72	0.658	0.511
Platelets (×10^9^/L), *M* (*P*_25_, *P*_75_)	212.43 ± 65.5	200.81 ± 55.51	1.31	0.192
Triglycerides (mmol/L), *M* (*P*_25_, *P*_75_)	1.52 (1.01, 2.09)	1.43 (0.98, 2.09)	0.043	0.965
Total cholesterol (mmol/L)	4.58 ± 1.06	4.45 ± 1.17	0.801	0.424
LDL-C (mmol/L)	2.99 ± 0.86	2.77 ± 0.92	1.756	0.081
HDL-C (mmol/L)	1.05 ± 0.2	1.18 ± 0.35	3.207	0.002^*∗*^
Creatinine (umol/L), *M* (*P*_25_, *P*_75_)	72.6 (61.45, 86.83)	66.9 (58.45, 81.4)	1.935	0.053
Uric acid (umol/L)	368.36 ± 98.98	383.33 ± 113	0.993	0.322
Glucose (mmol/L), *M* (*P*_25_, *P*_75_)	6.71 (5.45, 8.9)	5.15 (4.81, 5.82)	6.42	<0.001^*∗*^
LVEF, *M* (*P*_25_, *P*_75_)	60 (54.25, 62.75)	62 (60, 66.5)	3.735	<0.001^*∗*^
Serum YKL-40 (ng/dl) *M* (*P*_25_, *P*_75_)	1120.61 (624.6, 1537.62)	625.2 (351.79, 977.47)	4.9	<0.001^*∗*^
Serum TNF-*α* (pg/ml) *M* (*P*_25_, *P*_75_)	40.63 (38.31, 43.46)	30.91 (28.47, 34.87)	8.465	<0.001^*∗*^

*Note.* BMI: body mass index; LDL-C: low-density lipoprotein cholesterol; HDL-C: high-density lipoprotein cholesterol; LVEF: left ventricular ejection fraction; ^*∗*^*P* < 0.05. [Mean ± standard deviation, *M* (*P*25, *P*75), number of cases and percentage (%)].

**Table 2:** Logistic regression analysis of risk factors for STEMI.

	Univariate logistic regression analysis				Multivariate logistic regression analysis			
	*β*	Odds ratio	95% CI	*P*-value	*β*	Odds ratio	95% CI	*P*-value
Age	—	—	—	—	0.042	0.959	(0.903, 1.018)	0.168
Gender	—	—	—	—	1.714	5.552	(0.708, 43.554)	0.103
Hypertension	—	—	—	—	0.271	1.311	(0.334, 5.14)	0.698
BMI	0.089	0.915	(0.839, 0.997)	0.043^*∗*^	0.234	0.792	(0.606, 1.034)	0.087
Diabetes	1.152	0.316	(0.147, 0.68)	0.003^*∗*^	1.04	0.353	(0.066, 1.89)	0.224
Smoking	1.698	0.183	(0.098, 0.343)	<0.001^*∗*^	1.909	0.148	(0.031, 0.709)	0.017^*∗*^
Systolic pressure	0.018	0.982	(0.971, 0.994)	0.004^*∗*^	0.013	1.014	(0.968, 1.061)	0.568
Diastolic pressure	0.034	0.967	(0.948, 0.986)	0.001^*∗*^	0.051	0.951	(0.901, 1.003)	0.065
Neutrophils	0.662	1.939	(1.6, 2.349)	<0.001^*∗*^	0.903	2.467	(1.681, 3.621)	<0.001^*∗*^
HDL-C	1.702	0.182	(0.061, 0.548)	0.002^*∗*^	7.351	0.001	(0, 0.062)	0.002^*∗*^
Glucose	0.373	1.453	(1.21, 1.744)	<0.001^*∗*^	0.166	1.181	(0.924, 1.51)	0.184
LVEF	0.062	0.94	(0.901, 0.981)	0.004^*∗*^	0.019	0.981	(0.899, 1.07)	0.662
Serum YKL-40	0.001	1.001	(1.001, 1.002)	<0.001^*∗*^	0.002	0.998	(0.995, 1)	0.038^*∗*^
Serum TNF-*α*	0.254	1.29	(1.204, 1.381)	<0.001^*∗*^	0.403	1.496	(1.254, 1.784)	<0.001^*∗*^

*Note.* BMI: body mass index; HDL-C: high-density lipoprotein cholesterol; LVEF: left ventricular ejection fraction; ^*∗*^*P* < 0.05.

## Data Availability

The data supporting this study's findings are available from the corresponding author upon reasonable request.
